# Efficacy of Fragrance Types and Intervention Methods in Reducing Driver Fatigue and Modulating Emotional Development Assessed by HRV and Subjective Indicators

**DOI:** 10.3390/s25206450

**Published:** 2025-10-18

**Authors:** Zeping Chen, Qiang Liu, Bo Li, Jie Fu

**Affiliations:** 1 College of Intelligent Systems Engineering, Shenzhen Campus of Sun Yat-sen University, Shenzhen 518107, China; chenzp28@mail2.sysu.edu.cn (Z.C.); libo96@mail2.sysu.edu.cn (B.L.); fujie27@mail.sysu.edu.cn (J.F.); 2 Guangdong Marshell Electric Technology Company, Zhaoqing 526238, China; 3 Research and Development Center, Guangzhou Automobile Group Co., Ltd., Guangzhou 511434, China

**Keywords:** driving fatigue, emotional states, olfactory intervention, simulated driving experiments

## Abstract

Driver fatigue and negative emotions are significant factors contributing to traffic accidents. In-vehicle fragrance, as a fatigue intervention strategy, can help improve drivers’ mental and emotional states, preventing accidents. However, there is a lack of systematic research on how different fragrance types and release methods affect drivers’ fatigue and emotional development. Forty healthy drivers (mean age: 31 years, gender balanced) participated in this study. Participants were randomly assigned to two groups: one group tested three different fragrance types (HINOKI, GRASSY, YUZU), and the other group tested three fragrance release methods (CR: continuous release, IR: intermittent release, and PR: pulse release). All participants completed a simulated driving task under specified in-vehicle fragrance management conditions. Subjective fatigue ratings and emotional self-assessments (POMS) were used to assess changes in fatigue and emotions, and heart rate variability (HRV) was measured to evaluate physiological changes. Compared to no intervention, fragrance intervention significantly reduced drivers’ subjective fatigue ratings, with the continuous release mode showing a more pronounced reduction in fatigue scores. Fragrance intervention effectively improved heart rate variability, with significant differences observed between release modes. The fragrance intervention also had a significant effect on emotional ratings, notably increasing vigor and reducing negative emotions such as tension and anxiety. The impact of fragrance type on fatigue scores, HRV, and emotional ratings was limited, suggesting that the effectiveness of fragrance intervention may depend more on the intensity and release mode of the intervention rather than the fragrance type. Fragrance intervention effectively reduces driver fatigue and improves emotional states, with the continuous release mode showing the most significant effects. The findings of this study can provide valuable insights for customizing in-vehicle fragrance release strategies to alleviate fatigue and improve emotional well-being in individuals engaged in long-duration driving tasks, with significant implications for the management of drivers’ mental and psychological health.

## 1. Introduction

In modern society, long-distance driving has become a part of everyday life for many individuals, particularly in high-intensity work environments, where driver fatigue has become an increasingly serious issue. Driving fatigue not only affects the driver’s attention and reaction speed but also potentially leads to traffic accidents, posing significant risks to road safety. A significant association exists between collision accidents and driving fatigue. Approximately 10% to 20% of traffic accidents are caused by driving fatigue [1,2]. Therefore, the effective alleviation of driver fatigue and the improvement of the driving experience have become critical issues in the field of traffic safety.

With the advancement of technology, driving fatigue has been extensively studied. Mu et al. [3] selected 12 participants for a simulated driving experiment and developed a fatigue detection algorithm based on electroencephalogram (EEG) signals. Guo et al. [4] developed a multimodal attention network for driver fatigue detection based on EEG, electrodermal activity (EDA), and photoplethysmography (PPG) signals using the simulation driving experiment data of 14 participants. Although much research has been conducted on driving fatigue, most of it focuses on fatigue identification and detection [5,6]. The intervention and management of driving fatigue still requires further study.

Numerous studies have demonstrated that fragrance interventions, as a non-invasive psychophysiological approach, can effectively improve fatigue and mood by influencing both psychological states and physiological responses [7,8,9]. For instance, it pointed out that the scent characteristics of fragrances can directly connect to the emotional centers of the brain via the olfactory system, thereby inducing emotional regulation effects [10,11]. Many studies have shown that fragrances can significantly enhance drivers’ alertness and psychological states by modulating mood and alleviating stress [12,13,14]. However, research on the application of fragrances in driving fatigue intervention remains relatively limited. Specifically, there is a lack of systematic exploration of how factors such as fragrance type, intervention intensity, and release mode affect the psychophysiological states of drivers.

In the context of automobile driving, a driver’s physiological state, such as heart rate variability (HRV), is often used as an effective indicator of fatigue [15]. HRV reflects the balance of the autonomic nervous system and can reveal physiological changes in the driver due to fatigue during driving [5,16]. HRV is widely utilized as a key marker of fatigue in drivers and has been applied to investigate the relationship between drivers’ physiological states and driving behavior [17,18]. Persson et al. [19] utilized the real road data of 86 drivers who were either awake or in a state of sleep deprivation to explore the relationship between HRV and driver drowsiness. The results showed that sleepiness was associated with lowered heart rate and increased HRV. Recent studies have also indicated that HRV can not only reflect the level of fatigue but can also effectively predict drivers’ cognitive and emotional states [20,21]. These studies highlight HRV as a reliable physiological indicator for evaluating driver fatigue and its related emotional responses.

Moreover, a driver’s emotional state is one of the key factors influencing driving safety. Particularly in long-duration driving tasks, negative emotions cause the driver’s mental state to become unstable, thereby affecting their driving safety [22,23,24,25]. Research has shown that a driver’s emotional and psychological states directly impact driving responses and overall safety [26]. Negative emotions, such as anxiety and stress, can easily lead to slower reaction times and increase the risk of accidents [27]. Therefore, regulating the emotional state of drivers is of great significance in reducing human-caused traffic accidents [9,13]. Especially through fragrance intervention, it not only regulates the drivers’ emotions but also alleviates fatigue, which is conducive to further enhancing driving safety.

Thus, the present study aims to explore the effects of fragrance intervention on drivers’ subjective fatigue, HRV indicators, and emotional states, with a focus on how intervention intensity, fragrance type, and release mode modulate the psychophysiological states of drivers. Specifically, the study will examine the effects of different intervention intensities (no intervention and fragrance intervention), fragrance types (woody, herbal, and citrus scents), and fragrance release modes (continuous release, pulsed release, and intermittent release) on mitigating driver fatigue. The hypotheses of the study include that fragrance interventions can significantly improve driver fatigue and emotional states, and that different fragrance types and release modes will show significant differences in their modulation of HRV and emotional responses. The research aims to provide a theoretical basis for fragrance-based driving fatigue intervention strategies and explore the potential applications of fragrance as a safety management tool in driving.

## 2. Methodology

### 2.1. Participants

The study involved 40 healthy participants, who were randomly and equally divided into two groups for testing fragrance types and fragrance release modes. The fragrance type group consisted of 20 participants, with a balanced gender distribution, an average age of 31.3 years (SD = 6.74), an average driving experience of 6.74 years (SD = 4.2), and an average accumulated driving distance of 43,442 km. The fragrance release mode group also included 20 participants, with a balanced gender distribution, an average age of 31.9 years (SD = 7.84), an average driving experience of 6.05 years (SD = 4.34), and an average accumulated driving distance of 42,755 km. A self-reported screening procedure ensured that all participants were healthy and had normal olfactory function, with no history of respiratory diseases. According to the sleep survey report, 55% of the participants rated their sleep quality in the past week as excellent, 45% as normal, and none as poor. 80% of the participants reported falling asleep between 23:00 and 00:00 in the past week, 10% fell asleep before 23:00, and 10% fell asleep after 00:00 but before 01:00. 80% of the participants reported getting up between 07:00 and 09:00 in the past week (excluding the day of the experiment), and 20% got up between 06:00 and 07:00.

### 2.2. Experimental Equipment

**Figure 1:**
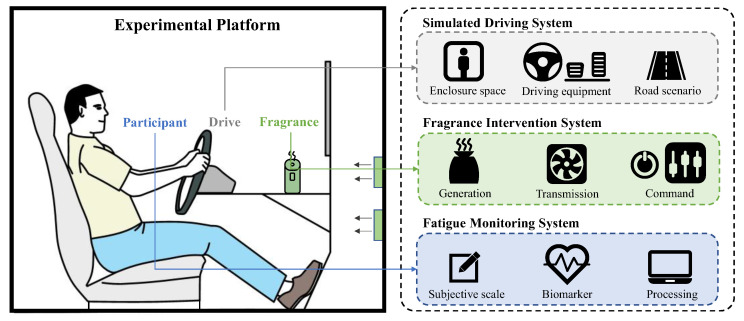
Enclosed driving simulator cockpit.

**Figure 2:**
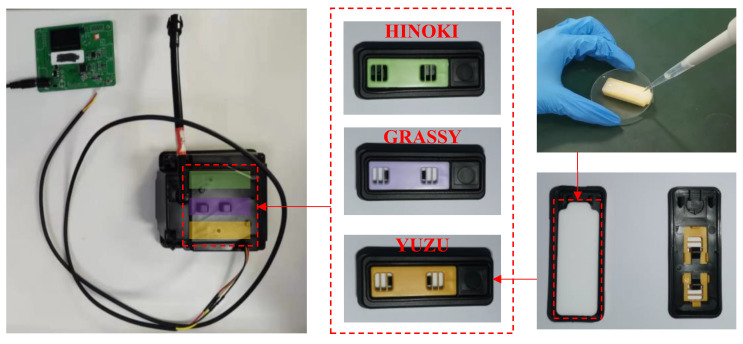
A solid fragrance generation device that can hold at most three different fragrances simultaneously. The fragrance essential oil is adsorbed via a white solid carrier.

The study was conducted in an enclosed driving simulator cockpit constructed with high-transmittance acrylic panels, integrating three functional modules, including a simulated driving system, a fragrance intervention system, and fatigue monitoring, as shown in Figure 1. Environmental parameters were maintained at 24–26°C with 40–50% relative humidity. A detailed description of the enclosed driving simulator cockpit can be found in Appendix A. The solid fragrance generation device was utilized to release fragrance, as shown in Figure 2. The released fragrance scent is transmitted to the driver through the cockpit fan.

### 2.3. Experimental Design

Three types of in-vehicle fragrances and three fragrance release modes were evaluated in this study. During the fragrance type test, the fragrance dispenser was set to a fixed release mode. During the fragrance release mode test, the dispenser was set to a fixed fragrance type. Specifically, the fragrance types were tested first, and a specific release mode CR was consistently used, with the combinations being “HINOKI and CR, GRASSY and CR, YUZU and CR”. Then the release modes were tested, and a specific fragrance called YUZU was consistently used, with the specific combinations being “YUZU and CR, YUZU and PR, YUZU and PR”.

#### 2.3.1. Fragrance Type

Three types of in-vehicle fragrances, named HINOKI, GRASSY, and YUZU, were assessed. These fragrances are from a certain automotive brand and claim to have anti-fatigue effects. They are formulated by mixing different base fragrance compounds. Due to commercial confidentiality, the fragrance formulas cannot be disclosed; however, the odor characteristics of these fragrances are publicly available, as shown in Table 1. These odor characteristics have also been found to alleviate fatigue and anxiety and enhance positive emotions in the research [28,29,30].

#### 2.3.2. Fragrance Release Mode

In this study, a fragrance dispenser, which was removed from a real vehicle, was used as the vehicle’s fragrance delivery system. The dispenser has three release modes: continuous release (CR), pulse release (PR), and intermittent release (IR). The release parameters for each mode are shown in Table 1. In the continuous release mode, the machine operates continuously. In the pulse release mode, the machine operates intermittently with short intervals between bursts. In the intermittent release mode, the machine operates intermittently with longer intervals. The operational pattern and the duration of the intervals directly influence the fragrance concentration within the vehicle, with the CR mode having the highest concentration, followed by PR, and IR having the lowest concentration. CR is the basic mode of the vehicle fragrance system; however, due to the phenomenon of olfactory adaptation [31], continuous odor stimulation can lead to a decrease in olfactory sensitivity. The PR and IR modes are designed to counteract this phenomenon. Based on periodic changes in stimulation, it is possible to extend the intervention effect.

### 2.4. Experimental Conditions and Procedures

The experimental protocol was approved by the university’s Institutional Review Board and conducted in accordance with the Declaration of Helsinki. Upon arrival, participants were informed about the study’s purpose, procedures, and potential risks, and they were asked to sign a written informed consent form.

**Figure A2:**
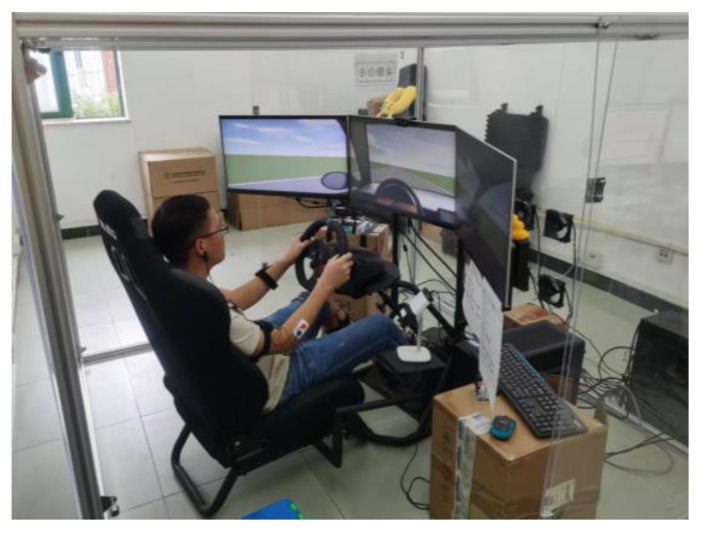
Participants’ experimental image records.

All driving tasks were conducted in the afternoon at 2:00 PM. Participants were instructed to avoid taking a nap on the day of the experiment to ensure they were in a fatigued state, as this would facilitate the onset of fatigue during the task. Participants were also instructed to refrain from consuming caffeine the day before the experiment and to remain awake during the 6:00 AM to 2:00 PM window on the test day. Each participant completed three monotonous driving tasks. The actual experimental scene is shown in Figure A2.

For the fragrance type test, participants were exposed to HINOKI fragrance during one driving task, GRASSY fragrance during another, and YUZU fragrance during the third. The order of fragrance types was randomized across participants, and they were blinded to which fragrance they were exposed to. All three fragrances were released after the participant had driven for 30 min. A similar procedure was followed for the fragrance release mode test.

**Figure 3:**
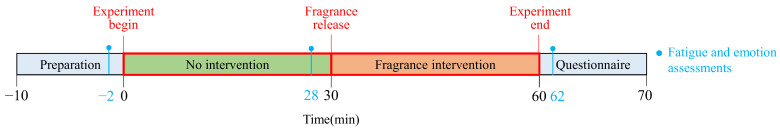
The experimental procedure of a single driving task for each participant. Each participant should complete three driving tasks.

The experimental procedure of the single driving task is shown in Figure 3. After the experiment began, participants performed the driving tasks in a closed driving simulator, using monotonous driving scenes to induce driver fatigue. The driving scenario consisted of a highway with a constant speed of 120 km/h and no other traffic, thus avoiding interference such as collisions.

After 30 min of driving, the experimenter activated the fragrance dispenser. The dispenser, containing solid fragrance blocks, was placed inside the cockpit approximately 1 m away from the participant’s face, with the nozzle directed towards the driver. Upon activation, the experimenter pressed a button to trigger the dispenser, which then released the designated fragrance according to the pre-programmed mode. The fragrance dispenser operated silently. Participants were informed that the study involved an evaluation of different scents, but they were unaware of the number of fragrances, the timing of their release, or the expected effects of these fragrances. After exposure to the fragrance, participants continued driving for another 30 min, at which point the experiment was concluded. The total duration of the experiment for each participant was 1 h. After the experiment ended, the participants filled out the questionnaire to report on the changes in fatigue and emotions during the experiment. Due to the fact that fatigue and emotion assessment will take approximately 1 min, the specific assessment time points for the 0 min (baseline) and 30 min will be advanced by two minutes, respectively. The specific assessment time point of 60 min will be delayed by 2 min and will be conducted with the questionnaire.

### 2.5. Measurement

#### 2.5.1. Subjective Fatigue

**Figure 4:**
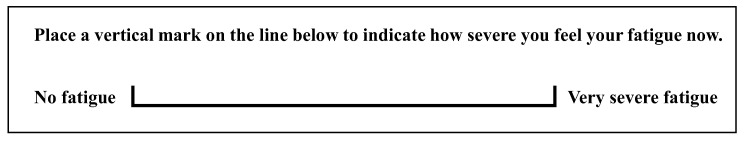
Visual analog fatigue scale.

Self-reported fatigue was assessed at three time points: before the driving task, at 30 min into the driving task, and after the driving task. Fatigue levels were measured using the Visual Analog Fatigue Scale (VAFS) [32]. The Visual Analog Scale (VAS) consists of a 10 cm horizontal line with written descriptions at each end (Figure 4). Participants were asked to mark a point on the line that they felt best represented their current state. The possible score range is from 0 to 1, and the score is measured in millimeters from the “no fatigue” end to the point indicated by the participant, using a ruler. The score is calculated by measuring the length of the line segment from the “no fatigue” point to the point indicated by the participant, divided by 100 mm. Higher VAFS scores indicate higher levels of fatigue.

#### 2.5.2. Electrocardiogram and HRV

**Figure 5:**
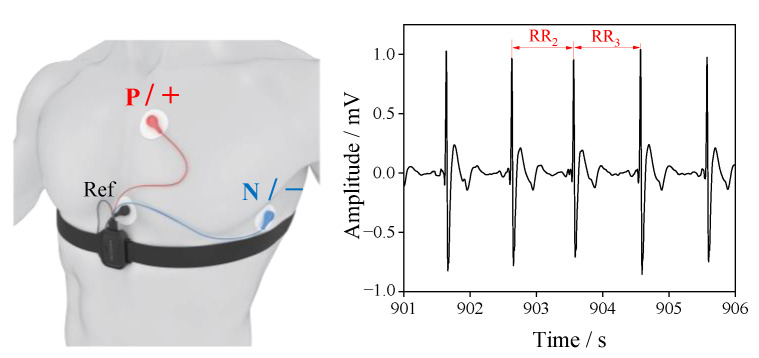
ECG sensor wearing guidance and ECG schematic diagram.

Electrocardiogram (ECG) signals were recorded using the ErgoLAB platform (Kingfar International Inc., Beijing, China). Electrocardiographic data were collected using a three-electrode configuration (Lead II) attached to the chest, recorded at 1024 Hz through the ErgoLAB system. The ECG sensor was secured to the chest using an elastic band, without interfering with normal driving (Figure 5). Raw ECG signals were bandpass-filtered (0.3–30 Hz) to remove noise, with NN interval extracted for Heart rate variability (HRV) analysis.

HRV is considered one of the most valuable non-invasive methods for assessing the autonomic nervous system (ANS). By consecutive heartbeats before and after the olfactory intervention, significant HRV indicators that can assess the arousal effects on fatigue were extracted. HRV analysis requires at least 2 min of data to ensure the accuracy of the analysis [33]. In this study, 5 min of data were used to calculate HRV to ensure accuracy. Specifically, the HRV analysis utilized 20–25 min blocks before the intervention and 55–60 min blocks after the intervention. The baseline was 5–10 min blocks. Overall, HR, SDNN, pNN50, and LF/HF were calculated according to the following formulas:

(1) $$HR=\frac{60}{{RR}_{i}}$$

where RR_i_ is i-th NN interval;

(2) $$SDNN=\sqrt{\frac{1}{N}\sum_{i=1}^{N}{\left({RR}_{i}-\mu \right)}^{2}}$$

where N is the number of NN intervals, and µ is the mean of all NN intervals;

(3) $$pNN50=\frac{{NN}_{50}}{N}$$

where NN_50_ was the number of NN intervals that differed by more than 50 ms;

(4) $$LF/HF=\frac{P_{LF}}{P_{HF}}$$

where P_LF_ was the power of the low frequency band (0.04 to 0.15 Hz), and where P_HF_ was the power of the high frequency band (0.15 to 0.4 Hz).

#### 2.5.3. Subjective Emotion

Subjective emotional data were collected at three time points: before the driving task (−2 min), after 30 min of driving (28 min), and after the driving task (62 min), using the POMS 2-A™ questionnaire (Q_A_, Q_B_, Q_C_). The Q_A_ data were used to assess participants’ baseline emotional levels prior to the experiment, the Q_B_ data were used to evaluate the impact of driving on participants’ emotions, and the Q_C_ data were used to evaluate the effect of fragrance management on participants’ emotional states. The POMS 2-A™ includes seven subscales and a Total Mood Disturbance (TMD) score. The seven subscales are: Tension–Anxiety (TA), Depression–Dejection (DD), Anger–Hostility (AH), Vigor–Activity (VA), Fatigue–Inertia (FI), Confusion–Bewilderment (CB), and “Friendliness” (FR). The Total Mood Disturbance (TMD) score is a global measure of emotional disturbance, psychological distress, or subjective well-being. It is defined as follows:

(5) $$TMD=\left(AH+CB+DD+FI+TA\right)-VA$$

For the positive scale VA, a higher score indicates better emotional status, while for the other five negative scales and the global indicator (TMD), a lower score indicates better emotional status. The TMD can be either negative or positive. The “Friendliness” (FR) subscale was omitted from this study because the TMD calculation only includes six emotions, excluding the FR.

### 2.6. Statistical Analysis

To minimize the effects of individual differences, changes in the dependent variables (subjective fatigue rating, HRV indicators, emotional rating) at different time points were compared individually for each participant. During each 60 min driving experiment, participants underwent a control condition in the first 30 min and a fragrance intervention in the latter 30 min. Therefore, the analysis of subjective fatigue and emotional scores utilized the 28 min and −2 min time points to calculate the data changes during the no intervention phase; and employed the 62 min and 28 min time points to calculate the data changes during the fragrance intervention phase. The HRV analysis employed 20–25 min blocks and 5–10 min blocks to calculate the data changes during the no intervention phase, and used 55–60 min blocks and 20–25 min blocks to calculate the data changes during the fragrance intervention phase.

In the fragrance type test, the two independent variables were fragrance type (3 levels: HINOKI, GRASSY, YUZU) and intervention intensity (2 levels: no intervention and fragrance intervention). The dependent variables were subjective fatigue rating, HRV indicators, and emotional rating. Fragrance type and intervention intensity were treated as fixed effects, while participants were treated as random effects.

In the fragrance release mode test, the two independent variables were fragrance release mode (3 levels: continuous, pulse, intermittent) and intervention intensity (2 levels: no intervention and fragrance intervention). The dependent variables were subjective fatigue rating, HRV indicators, and emotional rating. Fragrance release mode and intervention intensity were treated as fixed effects, while participants were treated as random effects.

Before performing hypothesis testing, the normality of the dependent variables was assessed using the Shapiro–Wilk and Kolmogorov–Smirnov tests. Hypothesis testing was conducted using Generalized Linear Mixed Models (GLMM). Any statistical significance was further investigated using post hoc multiple comparisons (Tukey’s HSD test) to address potential Type I error inflation due to multiple comparisons.

## 3. Results

### 3.1. Subjective Fatigue Rating

The subjective fatigue ratings of drivers increased under no intervention, whereas the ratings decreased under fragrance intervention, with significant differences observed between the two conditions (Table 2). The interaction between fragrance type and intervention intensity did not significantly affect subjective fatigue ratings, and no significant differences were found in fatigue ratings across the three fragrance types.

**Figure 6:**
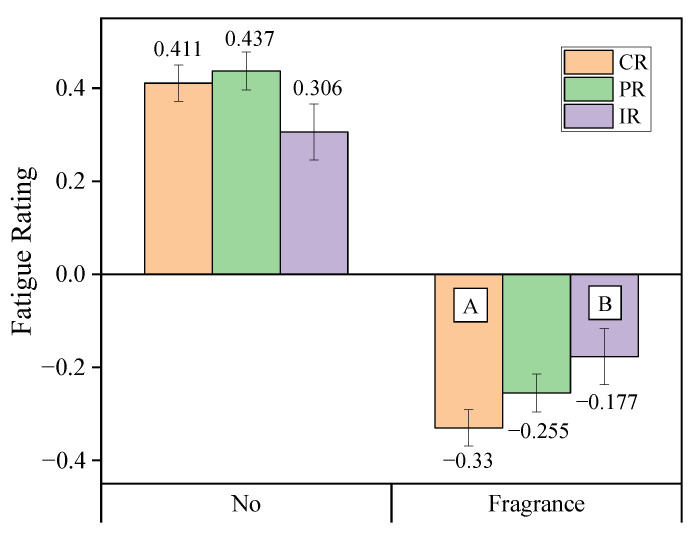
Interaction effect between release mode and intervention intensity on subjective fatigue rating. The letters A and B indicate a significant difference according to release modes.

In contrast, the interaction between release mode and intervention intensity significantly influenced subjective fatigue ratings (Figure 6). Under fragrance intervention, the continuous release mode (CR) resulted in significantly lower fatigue ratings compared to the intermittent release mode (IR). The pulse release (PR) mode’s fatigue rating showed no significant difference from the other two modes, and its fatigue rating was intermediate between the continuous and intermittent release modes.

### 3.2. HRV Indicators

The changes in HRV indicators were significantly influenced by intervention intensity. Specifically, without intervention, heart rate (HR) decreased, while LF/HF, SDNN, and pNN50 increased. Under fragrance intervention, the decrease in HR was significantly smaller compared to no intervention, and the increase in LF/HF and SDNN was smaller than without intervention, although no significant differences were found (Table 2).

The changes in HR were also significantly influenced by the release mode. In the continuous release mode, the reduction in HR was significantly greater than in the intermittent release mode (Table 3). The HR reduction in the pulse release mode showed no significant difference from the other two modes, with the reduction being intermediate between the continuous and intermittent modes.

Changes in HRV indicators were not significantly affected by fragrance type. Furthermore, there was no significant interaction between intervention intensity and fragrance type, nor between intervention intensity and release mode, in terms of HRV indicators.

### 3.3. Subjective Emotional Rating

In both experimental groups, fragrance intervention significantly reduced total negative emotions, with a significant reduction observed in all emotions except for vitality, and a significant increase in vitality/alertness (Table 2). This change was more pronounced in vitality, which increased by 66%; fatigue decreased by 64%, and depression decreased by 56%.

**Figure 7:**
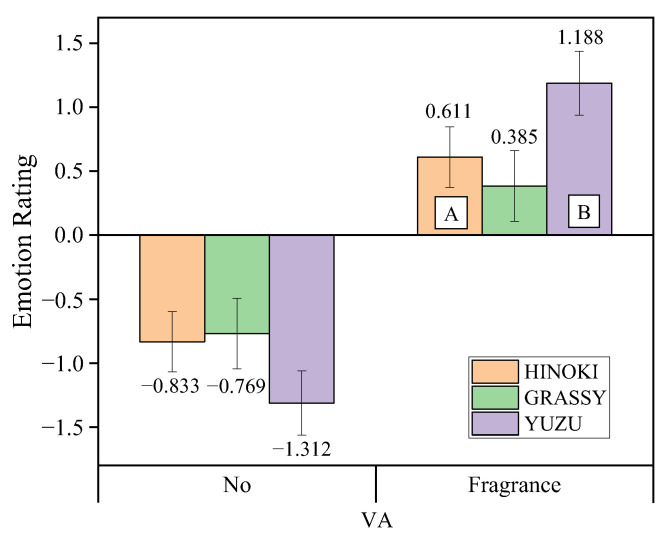
Interaction effect between fragrance type and intervention intensity on VA rating. The letters A and B indicate a significant difference according to fragrance types.

Fragrance type did not significantly affect TMD or the five negative emotions. In contrast, the interaction between fragrance type and intervention intensity significantly influenced Vigor-Activity (VA) ratings (Figure 7). Under fragrance intervention, the VA rating for YUZU was significantly higher than that for HINOKI, while GRASSY’s VA rating was lower than HINOKI’s, but there were no significant differences between GRASSY and the other two fragrances.

**Figure 8:**
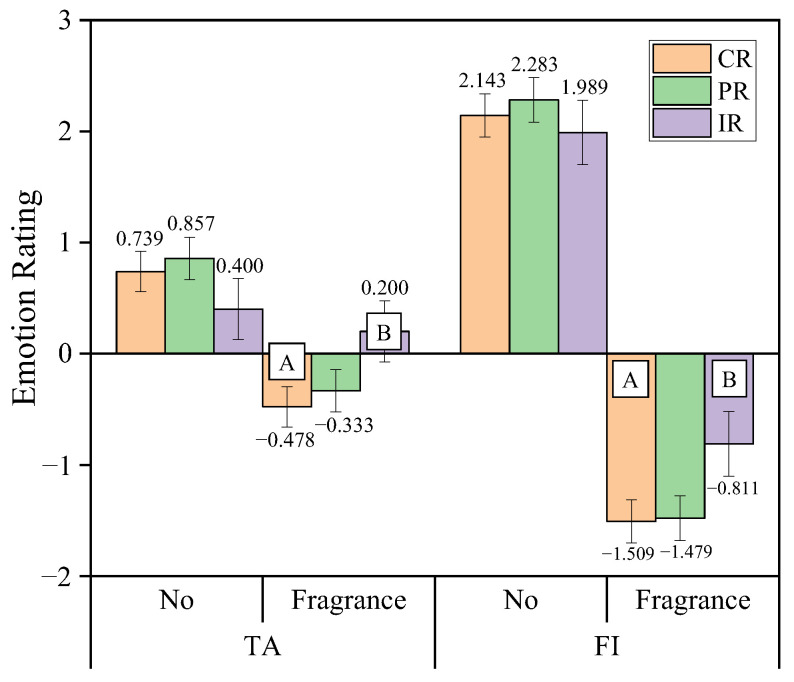
Interaction effect between release mode and intervention intensity on TA and FI rating. The letters A and B indicate a significant difference according to release modes.

Release mode and intervention intensity had a significant interaction effect on Tension-Anxiety (TA) and Fatigue-Inertia (FI) rating (Figure 8). Under fragrance intervention, the reduction in TA and FI ratings was significantly greater for the continuous release mode (CR) compared to the intermittent release mode (IR).

## 4. Discussion

This study evaluated the effects of intervention intensity (no and fragrance), three types of fragrance (HINOKI, GRASSY, YUZU), and three release modes (CR, PR, IR) on subjective fatigue ratings, HRV, and subjective emotional ratings during a simulated driving task. The results showed that fragrance intervention significantly reduced subjective fatigue scores, total emotional scores (TMD), and the extent of heart rate (HR) reduction. Compared to the other two modes, the continuous release mode (CR) significantly improved subjective fatigue, total emotional scores, and HRV indicators. Fragrance type had no significant effect on the above measures, except for vitality.

### 4.1. The Impact of Fragrance on Subjective Fatigue

The study results indicated that under no intervention, drivers’ subjective fatigue ratings significantly increased, while fragrance intervention led to a significant reduction in fatigue ratings. The interaction between fragrance type and intervention intensity had no significant effect on subjective fatigue ratings, and no significant differences in fatigue ratings were observed among the three fragrance types, suggesting that the different fragrances had similar effects on fatigue relief. As hypothesized, fragrance intervention improved subjective fatigue perception compared to no intervention. This finding aligns with previous studies, which have indicated that fragrance can positively influence fatigue perception through both physiological and psychological mechanisms [34,35]. However, significant differences were observed in the interaction between release mode and intervention intensity. The continuous release mode (CR) had a notably superior effect on subjective fatigue ratings compared to the intermittent release mode (IR). The continuous release mode provides a continuous fragrance stimulus to the driver, which may help alleviate fatigue during driving, whereas the intermittent release mode, due to its discontinuous fragrance delivery, may fail to maintain the same fatigue-relief effect. The fatigue ratings for the pulse release mode (PR) were intermediate between the continuous and intermittent modes, showing some alleviating effect but with no significant difference from the other two modes. This finding suggests that continuous fragrance release is more effective in regulating subjective fatigue, whereas intermittent release may be less effective due to the discontinuous nature of the stimuli.

### 4.2. The Physiological Effects of Fragrance

The changes in HRV indicators were significantly influenced by the intervention intensity. Specifically, under no intervention, drivers’ heart rate (HR) decreased, while the low-frequency/high-frequency ratio (LF/HF), standard deviation of NN intervals (SDNN), and 50% of NN intervals (pNN50) increased, reflecting a physiological response in which sympathetic nervous activity increases and parasympathetic nervous activity decreases under fatigue [36,37]. In contrast, under fragrance intervention, the reduction in HR was significantly less than that under no intervention. The increases in LF/HF and SDNN were also smaller than those observed without intervention, although these changes did not reach statistical significance. The alleviating effect of fragrance was evident in certain HRV indicators, although its intensity was not as pronounced as expected. This finding is consistent with previous research [38,39], which found that fragrance interventions could improve heart rate variability (HRV), especially in alleviating stress and anxiety. The release mode also had a significant impact on HR, with the continuous release mode showing a significantly smaller HR reduction compared to the intermittent release mode. This suggests that continuous release mode is more conducive to maintaining a stable heart rate and staying alert. The HR changes in the pulse release mode were intermediate between the continuous and intermittent modes, showing a moderate effect. This result indicated that continuous fragrance release has a more sustained effect on autonomic nervous system regulation, while intermittent release may lead to a diminished effect. Furthermore, the fragrance type has no significant influence on HRV. It is impossible to determine which fragrance is the most effective in improving HRV. The three fragrances have comparable effects on alleviating driving fatigue at the physiological level.

### 4.3. The Impact of Fragrance on Emotions

In terms of emotional ratings, fragrance intervention significantly improved drivers’ emotional states, particularly with a notable decrease in negative emotions. Specifically, fragrance intervention had the most significant effect on alleviating fatigue emotions, which decreased by 64%, while depressive emotions decreased by 56%, and vitality increased by 66% (Table 2). These results indicate that fragrance intervention effectively enhances drivers’ emotional states, especially by reducing fatigue and depression, and increasing vitality and alertness, thereby helping to maintain drivers’ mental state and vigilance. This finding aligns with previous research, which has shown that fragrance can not only relieve anxiety and stress but also effectively enhance vitality and alertness [40,41,42]. Fragrance type did not significantly affect the five negative emotions (including fatigue, depression, and tension), but there was a significant interaction between fragrance type and intervention intensity on vitality ratings. Specifically, citrus YUZU fragrance significantly increased vitality ratings, outperforming woody HINOKI fragrance, while herbal GRASSY fragrance had lower vitality ratings than HINOKI but showed no significant difference from the other two fragrances (Figure 7). This result may be related to the specific olfactory characteristics of the fragrances, as different scents may evoke different physiological and emotional responses, particularly in terms of enhancing vitality, with some fragrances being more effective. Similar studies have shown that citrus fragrances, due to their fresh and invigorating properties, can significantly improve mood vitality and alleviate negative emotional stress [28]. The citrus fragrance may primarily exert its effect through the cognitive-emotional pathway, directly improving the mental state of the participants rather than through the physiological level. For fatigue and tension emotions, the interaction between release mode and intervention intensity also showed a significant impact. Under fragrance intervention, the continuous release mode (CR) was more effective than the intermittent release mode (IR) in alleviating emotional tension and fatigue. The continuous release mode provides stable fragrance stimuli, helping to reduce drivers’ tension and fatigue, further confirming the effect of fragrance release mode on emotional improvement.

### 4.4. Limitations and Prospects

This study has several limitations. First, the study was conducted in a controlled laboratory environment with simulated driving tasks. As a result, the findings may not fully reflect real-world driving conditions, where drivers may experience greater psychological burdens.

Secondly, this study did not examine the influence of factors such as gender and age. Future research should recruit a larger and more diverse sample to systematically analyze these individual difference factors.

Thirdly, the 5 min HRV analysis window employed in this study may not be able to fully capture the transient physiological changes that occur within an extremely short period after the release of the fragrance, and it also fails to control for the potential influence of the cognitive state in the later stage of the driving task. Future studies could consider using shorter time windows or instantaneous HRV analysis methods to more accurately reveal the immediate effects of fragrance intervention.

Lastly, as a form of immediate sensory stimulation, fragrance intervention may have a noticeable short-term effect on improving fatigue and emotional states. However, the long-term effects of such interventions, and whether participants gradually become desensitized to the fragrance (olfactory adaptation), remain unclear. Future research could explore the long-term effects of fragrance interventions and potential adaptive mechanisms, or consider using periodic or varied fragrance interventions to maintain their effectiveness.

Furthermore, objective driving performance indicators (such as lane-keeping and steering variability) are crucial for assessing driving safety. Incorporating objective driving behavior indicators into the analysis is an important direction for future research. Despite the challenges that remain in the practical application of fragrance interventions for fatigue, research in this area is still of significant value. The results of this study can provide valuable insights for customizing in-vehicle fragrance release strategies and have important implications for managing drivers’ mental and psychological well-being.

## 5. Conclusions

The main finding of this study is that fragrance intervention can effectively reduce subjective driver fatigue during driving and significantly improve HRV and mood. Specifically, compared to no intervention, fragrance intervention significantly lowered subjective fatigue ratings, with a more pronounced reduction in fatigue scores under the continuous release mode. Fragrance intervention was also found to effectively improve HR, with significant differences observed across release modes. In comparison to the intermittent release mode, the continuous release mode resulted in smaller fluctuations in HR, indicating that continuous fragrance release is more effective in maintaining a stable physiological state. Fragrance intervention also had a significant impact on emotional ratings, notably improving vitality and significantly reducing negative emotions such as tension, contributing to greater mental alertness. Compared with the interval release mode, the scores for tension and fatigue in the continuous release mode were significantly lower. These findings indicate that fragrance can effectively alleviate driver fatigue and improve their mood by influencing their physiology and emotions, especially in the continuous release mode.

This study also found that the differences in the fatigue-reducing effect of fragrance intervention were more closely related to the intensity of the intervention and the release mode, rather than the fragrance type. All three fragrances can effectively reduce the subjective fatigue score and improve the HRV indicators; however, the differences among them are relatively small, making it impossible to make a physiology-based judgment on which type of fragrance has the best effect. The differences among the three fragrances primarily lie in emotional ratings, which could be related to the odor characteristics of the fragrances. Compared to the other two fragrances (the woody HINOKI and herbal GRASSY), the citrus YUZU fragrance notably enhanced vitality.

The findings of this study can provide valuable insights for customizing in-vehicle fragrance release strategies to alleviate fatigue and improve emotional well-being in individuals engaged in long-duration driving tasks. This has significant implications for the management of drivers’ mental and psychological health.

## Figures and Tables

**Table 1:** Description of the three fragrance types and the three fragrance release modes.

Fragrance Type	Description	Release Mode	Description
HINOKI	Wood notes with fresh, quiet wood aromas	CR	Work continuously
GRASSY	Herbaceous notes, like freshly cut grass, green leaves, or plants	PR	Work intermittently for a short period of time, work for 20 s, stop for 20 s
YUZU	Citrus notes with fresh, slightly sweet, and sour notes characteristic of grapefruit	IR	Long time intermittent work, work 20 s, stop 100 s

**Table 2:** Mean (Standard Error, SE) comparisons of the dependent variables (subjective fatigue rating, HRV indicators, emotional rating) by no intervention and fragrance intervention. The calculation of the percentage change value is based on the premise that the baseline value is 0. *p*-values were obtained from a GLMM. Asterisks denote statistical significance (*p* < 0.05).

Parameters	Intervention Intensity			F	*p*-Value
	No Intervention	Fragrance Intervention	Percentage Change		
Fatigue Rating	0.442 (0.019)	−0.307 (0.019)	−69.46%	753.767	<0.001 *
HR	−2.087 (0.580)	−1.256 (0.580)	−60.18%	4.678	0.032 *
LF/HF	1.017 (0.459)	0.314 (0.459)	30.88%	1.513	0.220
SDNN	7.594 (1.493)	5.348 (1.493)	70.42%	1.798	0.181
pNN50	1.350 (0.697)	2.819 (0.697)	208.81%	2.631	0.106
TMD	5.307 (0.340)	−3.158 (0.340)	−59.51%	310.778	<0.001 *
TA	0.673 (0.088)	−0.347 (0.088)	−51.56%	67.729	<0.001 *
AH	0.297 (0.056)	−0.119 (0.056)	−40.07%	27.434	<0.001 *
DD	0.446 (0.069)	−0.248 (0.069)	−55.61%	50.662	<0.001 *
FI	2.337 (0.092)	−1.505 (0.092)	−64.40%	877.808	<0.001 *
CB	0.624 (0.088)	−0.327 (0.088)	−52.40%	58.522	<0.001 *
VA	−0.931 (0.095)	0.614 (0.095)	65.95%	133.524	<0.001 *

**Table 3:** Mean (SE) comparisons of HRV indicators by fragrance type and by release mode. The letters A and B in superscript indicate a significant difference across the intervention conditions. *p*-values were obtained from a GLMM. Asterisks denote statistical significance (*p* < 0.05).

Parameters	Fragrance Type				Release Mode			
	HINOKI	GRASSY	YUZU	*p*-Value	CR	PR	IR	*p*-Value
HR	−0.808 (0.620)	−1.401 (0.697)	−1.568 (0.646)	0.478	−1.502 (0.384) ^A^	−2.392 (0.399)	−3.234 (0.560) ^B^	0.023 *
LF/HF	0.341 (0.480)	0.561 (0.521)	1.095 (0.493)	0.182	0.151 (0.250)	0.181 (0.259)	−0.198 (0.363)	0.645
SDNN	5.339 (2.618)	6.365 (3.021)	7.364 (2.755)	0.821	6.269 (1.949)	6.774 (1.998)	7.390 (2.646)	0.920
pNN50	1.426 (1.167)	1.279 (1.345)	1.633 (1.228)	0.975	3.453 (1.245)	2.115 (1.266)	3.106 (1.574)	0.503

**Figure A1:**
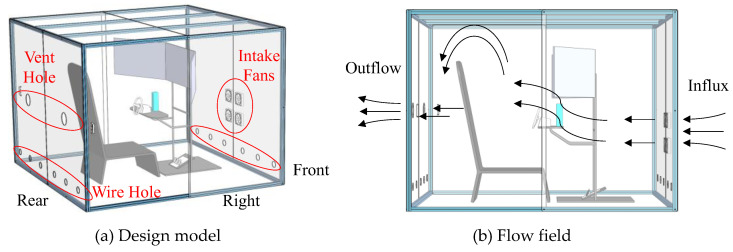
The transparent rectangular enclosure.

## Data Availability

The data included in this study are available upon request by contacting the corresponding author. The data are not publicly available because of ethical restrictions.

## Appendix A

The current setups for simulated driving environments used in olfactory intervention studies still require improvement. In one study [12], participants were required to wear a mask-like olfactory stimulation device during the experiment. In another study [13], the tube from the nebulizer containing the fragrance was attached to the participant’s chest, with the tube opening directed towards the nose. These studies employed odor release methods close to the nose; however, these methods do not apply to actual driving and may introduce additional distractions. Therefore, to better reconstruct the diffusion of odors in an actual vehicle, an enclosed simulated driving environment was setup in this study, as shown in Figure A1.

The transparent rectangular enclosure is composed of a rectangular aluminum alloy frame and transparent acrylic panels. Except for the bottom, the other five sides of the rectangular enclosure are covered with acrylic panels. The acrylic panels on the left and right sides are equipped with hinges and handles, allowing them to be opened and closed. The internal volume of the enclosure is approximately 5.6 m^3^, referencing the SUV internal volume of 5–6 m^3^. The front and rear sides of the enclosure are equipped with four and two vent holes, respectively. The four vent holes on the front side are fitted with fans for active air intake. The fan speed is adjustable, and the airflow rate range when all four fans are operating simultaneously is between 238 and 421.6 m^3^/h, referencing the car air conditioning wind speed of 200–500 m^3^/h. The bottom of the enclosure has two rows of wire holes for the entry of power and data cables.

**Table A1:** Parameters of the enclosure and fan.

Parameter	Enclosure	Parameter	Fan
Length (m)	1.96	Rated voltage (VDC)	12
Width (m)	1.96	Rated current (A)	0.12
Height (m)	1.46	Power (W)	1.44
Volume (m^3^)	5.6	Rotational Speed (rpm)	650–2000
Diameter of vent hole (mm)	115	Airflow rate (m^3^/h)	59.5–105.4
Diameter of wire hole (mm)	60	Noise (dBA)	5.5–22
